# All‐*Trans*‐Retinoic Acid‐Adjuvanted mRNA Vaccine Induces Mucosal Anti‐Tumor Immune Responses for Treating Colorectal Cancer

**DOI:** 10.1002/advs.202309770

**Published:** 2024-03-25

**Authors:** Wei Li, Yijia Li, Jingjiao Li, Junli Meng, Ziqiong Jiang, Chen Yang, Yixing Wen, Shuai Liu, Xingdi Cheng, Shiwei Mi, Yuanyuan zhao, Lei Miao, Xueguang Lu

**Affiliations:** ^1^ Beijing National Laboratory for Molecular Sciences CAS Key Laboratory of Colloid Interface and Chemical Thermodynamics Institute of Chemistry Chinese Academy of Sciences Beijing 100190 China; ^2^ University of Chinese Academy of Sciences Beijing 100049 China; ^3^ State Key Laboratory of Natural and Biomimetic Drugs School of Pharmaceutical Sciences Peking University Beijing 100191 China; ^4^ Beijing Key Laboratory of Molecular Pharmaceutics and New Drug Delivery System School of Pharmaceutical Sciences Peking University Beijing 100191 China

**Keywords:** all‐trans‐retinoic acid, cancer vaccine, colorectal cancer, mRNA, mucosal immune response

## Abstract

Messenger RNA (mRNA) cancer vaccines are a new class of immunotherapies that can activate the immune system to recognize and destroy cancer cells. However, their effectiveness in treating colorectal cancer located on the mucosal surface of the gut is limited due to the insufficient activation of mucosal immune response and inadequate infiltration of cytotoxic T cells into tumors. To address this issue, a new mRNA cancer vaccine is developed that can stimulate mucosal immune responses in the gut by co‐delivering all‐*trans*‐retinoic acid (ATRA) and mRNA using lipid nanoparticle (LNP). The incorporation of ATRA has not only improved the mRNA transfection efficiency of LNP but also induced high expression of gut‐homing receptors on vaccine‐activated T cells. Additionally, the use of LNP improves the aqueous solubility of ATRA, eliminating the need for toxic solvents to administer ATRA. Upon intramuscular injections, ATRA‐adjuvanted mRNA‐LNP significantly increase the infiltration of antigen‐specific, cytotoxic T cells in the lamina propria of the intestine, mesenteric lymph nodes, and orthotopic colorectal tumors, resulting in significantly improved tumor inhibition and prolonged animal survival compared to conventional mRNA‐LNP without ATRA. Overall, this study provides a promising approach for improving the therapeutic efficacy of mRNA cancer vaccines against colorectal cancer.

## Graphical abstract

A new mRNA cancer vaccine is developed that can stimulate mucosal anti‐tumor immune responses in the gut by co‐delivering all‐*trans* retinoic acid (ATRA) and mRNA using lipid nanoparticles (LNP). ATRA adjuvanted LNP significantly increase the infiltration of antigen‐specific, cytotoxic T cells in orthotopic colorectal tumors compared with LNP without ATRA, therefore effectively inhibiting tumor growth.

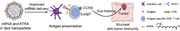

## 1 Introduction

Colorectal cancer has the third‐highest incidence and the second‐highest mortality among all cancers.^[^ ^1^ ^]^ The primary treatments for colorectal cancer are surgical resection, chemotherapy, radiotherapy, and targeted therapy. The recurrence rate after these treatments remains high.^[^ ^2^ ^]^ Cancer immunotherapy represents a new generation of cancer treatment and holds great promise for treating colorectal cancer.^[^ ^3^ ^]^ For example, immune checkpoint blockade therapy has been approved to treat a small portion of colorectal cancer patients that have high microsatellite instability or mismatch repair deficiency.^[^ ^4^ ^]^ However, the low response rate and limited applicability of immune checkpoint blockade therapy have restricted its clinical benefit.^[^ ^5^, ^6^, ^7^ ^]^ As another important class of immunotherapeutics, cancer vaccine leverages the host's own immune system to produce cytotoxic T cells that can specifically attack and destroy cancer cells.^[^ ^8^ ^]^ Cancer vaccines based on cells, viral vectors, and peptides have shown good efficacy and entered clinical stages.^[^ ^9^ ^]^ Recently, cancer vaccines based on messenger RNA (mRNA) have drawn significant attention following the approval of the two mRNA vaccines against coronavirus disease 2019 (COVID‐19).^[^ ^10^, ^11^ ^]^ mRNA vaccines possess several advantages, including their ability to induce both humoral and cellular immune responses, their capacity for rapid development and manufacturing, and their potential as personalized cancer vaccines.^[^ ^12^ ^]^ Early attempts to develop mRNA cancer vaccines were impeded by the high immunogenicity, instability, and poor cellular uptake of mRNA. The advancement of mRNA modification and delivery technologies has opened the door for the clinical use of mRNA cancer vaccines.^[^ ^13^ ^]^

Among different classes of delivery materials, lipid nanoparticle (LNP) is currently the only clinically approved delivery system for mRNA.^[^ ^14^, ^15^, ^16^ ^]^ LNP is composed of ionizable lipid, helper lipid, cholesterol, and polyethylene glycol (PEG)‐lipid, and has been used as a delivery vehicle for several mRNA cancer vaccines that have entered clinical trials.^[^ ^17^, ^18^, ^19^ ^]^ These vaccines are generally administered through intramuscular or intravenous injections, which activate antigen‐presenting cells such as dendritic cells (DCs) in draining lymph nodes and trigger the production of antigen‐specific cytotoxic T cells.^[^ ^20^ ^]^ However, because of the anatomic compartmentalization and functional distances, these activated DCs and T cells have limited efficacy in treating colorectal cancer that occurs on the mucosal surface (stage 0) and gradually grows to the lining (stage I) and through the wall of the intestine (stage II), since they barely traffic to the gut. To overcome this issue, oral or rectal immunizations have been proposed as promising methods to elicit mucosal immune responses in the gut.^[^ ^21^, ^22^, ^23^ ^]^ Nonetheless, poor penetration efficacy across the mucus layer and the instability of antigens and carrier systems in the gastrointestinal tract remain challenging obstacles to overcome.^[^ ^24^ ^]^ Thus, mRNA vaccines that can effectively activate anti‐tumor immune responses on the mucosal surface of the gut through parenteral administration are still very much needed to improve the efficacy of treating colorectal cancer.

All‐*trans*‐retinoic acid (ATRA) is a metabolite of vitamin A found in the diet that plays a vital role in lymphocyte trafficking and mucosal immunity.^[^ ^25^, ^26^, ^27^ ^]^ During antigen presentation, ATRA imprints activated T cells with gut tropism by inducing their expression of chemokine receptor type 9 (CCR9) and α4β7 integrin. CCR9 and α4β7 bind to chemokine ligand 25 (CCL25) expressed by intestinal epithelial cells and mucosal addressin cell adhesion molecule‐1 (MAdCAM‐1) expressed in gut‐associated lymphoid tissues, respectively.^[^ ^28^, ^29^, ^30^, ^31^ ^]^ Therefore, the parenteral administration of vaccines with ATRA represents a promising strategy to activate mucosal immune responses. ATRA is a hydrophobic molecule that also requires a delivery vehicle to improve its aqueous solubility for safe administration. Several studies have shown that intramuscular or subcutaneous injections of protein antigens and ATRA in separate or the same delivery vehicles elicited both systemic and mucosal immune responses.^[^ ^32^, ^33^, ^34^, ^35^ ^]^ For example, Dietrich et al. utilized two liposome formulations to separately deliver ATRA and recombinant chlamydia antigen.^[^ ^36^ ^]^ Antigen‐specific intestinal IgA response was observed when administering a high dose of ATRA (≈300 µg). Recently, Sun et al. achieved co‐delivery of antigen and ATRA by mesoporous silica nanoparticles, which activated mucosal immune response with a reduced dose of ATRA (50 µg) and protected immunized mice against the challenge of *S.Typhimurium*.^[^ ^37^ ^]^ Despite these progresses, whether ATRA could improve the therapeutic efficacy of mRNA cancer vaccines against colorectal cancer has not been explored. Additionally, current delivery systems for ATRA and protein antigens require multi‐step synthesis and still face challenges on scale‐up production and potential safety concerns in further translational studies.

**Figure 1:**
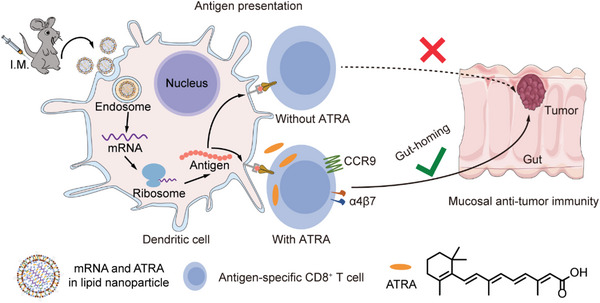
Schematic representation showing the mechanism of ATRA‐LNP on activating anti‐tumor immune responses in the gut. Specifically, ATRA‐LNP encapsulating mRNA vaccine and ATRA was administered through intramuscular injections (I.M.). ATRA‐LNP was then taken up by antigen‐presenting cells such as dendritic cells and expressed encoded tumor antigen. During antigen presentation, the presence of ATRA triggered the expression of gut‐homing receptors, CCR9, and α4β7, on activated T cells, which could then infiltrate into orthotopic colorectal tumors and kill tumor cells.

Herein, we developed an LNP formulation for the co‐delivery of mRNA cancer vaccine and ATRA through one‐step preparation. Interestingly, the incorporation of ATRA improves the mRNA delivery efficacy of LNP both in vitro and in vivo. ATRA in LNP also induced high expression of gut‐homing receptors CCR9 and α4β7 on activated T cells, thereby imprinting these T cells with gut tropism and inducing a mucosal immune response. In contrast, LNP without ATRA failed to trigger antigen‐specific T‐cell responses in the gut (**Figure** **1**). We further demonstrated that ATRA‐loaded LNP encapsulating a model mRNA cancer antigen effectively activated dendritic cells in the draining lymph nodes, elicited strong systemic T‐cell responses, and significantly increased the infiltration of antigen‐specific, cytotoxic T cells in orthotopic colorectal tumors, compared with the same LNP formulation without ATRA. The enhanced mucosal anti‐tumor immune response resulted in greatly improved tumor inhibition against an orthotopic colorectal tumor model in mice. Based on the simple production process, good therapeutic efficacy, and clinical safety demonstrated previously, our approach of using LNP to co‐deliver ATRA and mRNA cancer vaccine could offer a new solution for treating colorectal cancer.

## 2 Results and Discussion

### 2.1 Preparation and Characterization of LNPs

**Figure 2:**
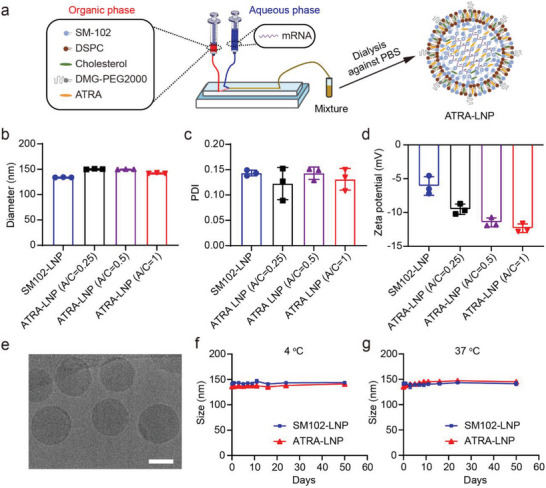
a) Schematic representation of the preparation process of ATRA‐LNP. The hydrodynamic diameters b), polydispersity indexes (PDI) c), and zeta potentials d) of ATRA‐LNP containing different amounts of ATRA. A/C represents the molar ratio of ATRA to cholesterol. SM102‐LNP without ATRA was used as a control. e) A representative cryo‐EM image of ATRA‐LNP. The scale bar is 50 nm. f and g) The hydrodynamic diameters of ATRA‐LNP and SM102‐LNP after storage a 4 and 37 °C. Data are shown as mean ± SD (standard deviation). n = 3 technical replicates.

The chemical structure of ATRA, which comprises a hydrophobic alkyl chain and a hydrophilic carboxylic acid end group, is akin to the structure of lipids. Moreover, ATRA has a molecular mass (300.4 Da) that is comparable to cholesterol (387.6 Da). Based on these similarities, we hypothesized that ATRA could be directly incorporated into the lipid bilayers of LNP during the self‐assembly of lipids, cholesterol, and mRNA. To test this hypothesis, we prepared an LNP formulation of the Moderna COVID‐19 vaccine, which was comprised of heptadecan‐9‐yl 8‐((2‐hydroxyethyl) (6‐oxo‐6‐(undecyloxy) hexyl) amino) octanoate) (SM‐102) as ionizable lipid, 1,2‐distearoyl‐sn‐glycero‐3‐phosphochline (DSPC) as helper lipid, cholesterol, and 1,2‐dimyristoyl‐rac‐glycero‐3‐methoxy polyethylene glycol‐2000 (DMG‐PEG2000), at a molar ratio of 50:10:38.5:1.5, respectively. The ratio of nitrogen in ionizable lipid to phosphorus in mRNA (N/P) was 5.67. We then dissolved ATRA in the ethanol phase containing all lipids before mixing them with firefly luciferase mRNA (mLuc) through a microfluidic device (**Figure** **2**a). We tested different amounts of ATRA to determine its loading capacity in LNPs, based on its molar ratio to cholesterol (A/C).

Dynamic light scattering measurements showed that LNPs with different amounts of ATRA (ATRA‐LNP) were all successfully fabricated with nearly the same hydrodynamic diameters and polydispersity index (PDI) as those of the LNP without ATRA (SM102‐LNP) (Figure 2b,c). The incorporation of ATRA did not affect the mRNA encapsulation efficiency of LNPs (Table S1, Supporting Information). Zeta‐potential measurements showed that the surface charge of ATRA‐LNPs was more negative than that of SM102‐LNP and decreased with an increasing amount of ATRA (Figure 2d). These results indicate that the hydrophilic carboxyl group of ATRA may be present on the surface of LNP, thus decreasing the surface potential. This observation in turn suggests the successful encapsulation of ATRA into LNP. To maximize the efficacy of ATRA‐LNP on inducing mucosal immune responses, we selected ATRA‐LNP with the highest ATRA amount (A/C = 1) for further investigation. The encapsulation efficiency of ATRA in LNP is ≈76% as determined by high‐performance liquid chromatography (HPLC, Figure S1, Supporting Information). To improve the relative mass ratio of ATRA to mRNA, we increased the N/P ratio from 5.67:1 to 15:1 while keeping the relative amounts of ATRA to lipids the same (Table S2, Supporting Information). ATRA‐LNP with a higher N/P ratio maintained nearly identical size, PDI, and mRNA encapsulation efficiency (Figure S2, Supporting Information). ATRA‐LNP exhibited a spherical morphology as shown in the cryogenic electron microscopy (Cryo‐EM) image (Figure 2e). ATRA‐LNP maintained its colloidal stability for at least 50 days at both 4 and 37 °C (Figure 2f,g). Collectively, these data demonstrated the successful one‐step fabrication of ATRA‐LNP with high encapsulation efficiencies for both mRNA and ATRA.

### 2.2 The mRNA Delivery Efficiency of ATRA‐LNP

**Figure 3:**
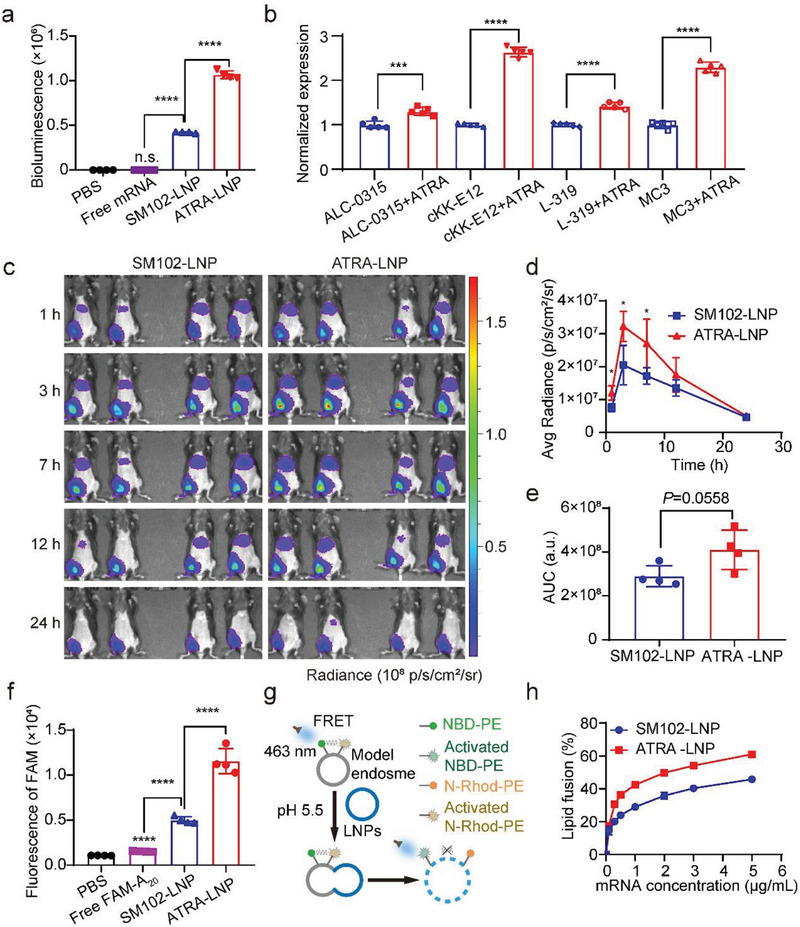
a) Transfection efficiency of free mRNA, SM102‐LNP, and ATRA‐LNP (N/P = 15) in DC2.4 cells (n = 4). b) Transfection efficiency of LNPs that are formulated with different ionizable lipids (N/P = 15) in DC2.4 cells (n = 5). c) IVIS images of mice receiving intramuscular injections of PBS, SM102‐LNP, or ATRA‐LNP (1 µg of mLuc per mouse, n = 4 biologically independent samples). d and e) Quantification of bioluminescence signals of mice and the area under the curve of luminescence signals over time (n = 4 biologically independent samples). f) Flow cytometry analysis of DC2.4 cells treated with free FAM‐A_20_, FAM‐A_20_‐loaded SM102‐LNP, or ATRA‐LNP (n = 4). g) Schematics view of the membrane fusion assay using fluorescence resonance energy transfer (FRET). h) The fusion of SM102‐LNP or ATRA‐LNP with model endosomes at different mRNA concentrations at pH 5.5 (n = 4). Data are shown as mean ± SD. Statistical analysis was calculated using the unpaired two‐tailed Student's *t*‐test b and e), one‐way analysis of variance (ANOVA) (a and f), or two‐way ANOVA d) with Tukey's multiple comparisons test: ^*^ *p* < 0.05, ^**^ *p* <0.01, ^***^ *p* <0.001, ^****^ *p* <0.0001, n.s. represents not statistically significant.

Previous studies have shown that the composition of lipid components in LNP significantly affects the mRNA delivery efficiency of LNP. To study whether ATRA affects the mRNA delivery of LNP, we incubated ATRA‐LNP encapsulating mLuc with DC2.4 cells and measured the expression of luciferase. Free mRNA and SM102‐LNP were used as controls. As shown in **Figure** **3**a, free mRNA could barely transfect DC2.4 cells with a nearly undetectable luciferase signal. SM102‐LNP exhibited very high mRNA delivery efficiency. Surprisingly, ATRA‐LNP showed ≈2.5 times the transfection efficiency of SM102‐LNP. To further verify that ATRA improved mRNA delivery of LNP, we incorporated ATRA into different LNP formulations that were formulated with different commonly used ionizable lipids including ALC‐0315, cKK‐E12, L‐319, and DLin‐MC3‐DMA (MC‐3). The ratio of ionizable lipids to helper lipids in these formulations is the same as SM102‐LNP. Figure 3b shows that the incorporation of ATRA improved mRNA expression for all tested LNPs, suggesting adding ATRA is a general strategy for improving the mRNA delivery efficiency of LNPs.

We next evaluated the mRNA delivery efficiency of ATRA‐LNP in vivo. Female C57BL/6 mice were administered with SM102‐LNP or ATRA‐LNP through intramuscular injection. The expression of mLuc was monitored by in vivo imaging system. As shown in Figure 3c, both SM102‐LNP and ATRA‐LNP exhibit strong luciferase signals at 1 h post‐injection, suggesting rapid cellular uptake, and mRNA expression. The expression of luciferase increased from 1 to 3 h and then gradually decreased over 24 h. Both LNPs had strong mRNA expression at injection sites. mRNA expression in the liver was also observed (Figure S3, Supporting Information), even though the magnitude was significantly lower than that in the muscle. These data suggest that a portion of LNPs enter the bloodstream after intramuscular injection and accumulate in the liver. Such off‐target expression in the liver is normally observed for LNPs following intramuscular injections^[^ ^38^, ^39^ ^]^ Quantification of IVIS images revealed that ATRA‐LNP exhibited higher mRNA expression than SM102‐LNP at all tested time points (Figure 3d). The average area under the curve (AUC) of ATRA‐LNP is ≈42% higher than that of SM102‐LNP (Figure 3e), suggesting that ATRA‐LNP also improved the mRNA expression of LNP in vivo.

We next investigate the mechanism of ATRA for improving LNP delivery. The mRNA delivery efficacy of LNP is largely determined by its cellular uptake and endosomal escape efficiencies. To study the cellular uptake of LNPs, we first prepared ATRA‐LNP and SM102‐LNP encapsulating fluorescein amidites (FAM)‐labeled oligonucleotides (FAM‐A_20_) and evaluated their cellular uptake using DC2.4 cells. ATRA‐LNP exhibited ≈1.7 times higher cellular uptake compared to SM102‐LNP (Figure 3f). To evaluate the endosomal escape of LNPs, we prepared a model endosome using two fluorescent dye‐labeled lipids, N‐4‐nitrobenzo‐2‐oxa‐1,3‐diazole‐phosphatidylethanolamine (NBD‐PE), and 1,2‐dioleoyl‐sn‐glycero‐3‐phosphoethanolamine‐N‐ (lissamine rhodamine B sulfonyl) ammonium salt (N‐Rhod‐PE). The Rhod fluorescence is activated by the emission of NBD through fluorescence resonance energy transfer (FRET). When the LNPs merge with these model liposomes, the Rhod fluorescence will decrease due to the increased distance between Rhod and NBD (Figure 3g). As shown in Figure 3h, ATRA‐LNP exhibited higher fusion ability compared to that of SM102‐LNP. Collectively, these results indicate that the incorporation of ATRA into LNP improved its cellular uptake and endosomal escape, leading to enhanced mRNA expression. This finding could have promising implications for the development of more effective LNP‐based mRNA therapies.

### 2.3 ATRA‐LNP Enhances Expression of Gut‐Homing Receptors on T Cells

**Figure 4:**
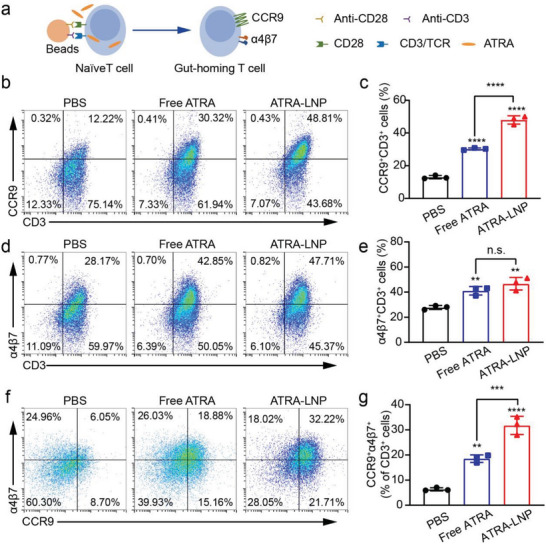
a) Schematic view of the in vitro T cell activation assay. Isolated CD8^+^ T cells from the spleen of mice were activated by anti‐CD3/CD28 antibodies‐coated beads. ATRA‐LNP in PBS or free ATRA in DMSO were incubated with T cells during activation. The expressions of CCR9 and α4β7 were quantified by flow cytometry. Representative flow cytometry plots and quantification analysis of CCR9^+^CD3^+^ T cells (b and c), α4β7^+^CD3^+^ T cells d and e), and CCR9^+^α4β7^+^CD3^+^ T cells f and g). n = 3 technical replicates. Data are shown as mean ± SD. Statistical analysis was calculated using one‐way ANOVA and Tukey's multiple comparisons tests: ^**^ *p* <0.01, ^***^ *p* <0.001, ^****^ *p* <0.0001, n.s. represents not statistically significant.

After demonstrating that ATRA enhanced the transfection efficacy of LNP, we investigated the function of ATRA in LNP. The presence of ATRA during T cell activation was shown to induce expression of CCR9 and α4β7, thus imprinting T cells with gut tropism. To mimic the antigen‐presenting process, we isolated T cells from the spleen of female C57BL/6 mice and stimulated T cells with anti‐CD3/CD28 antibodies‐coated magnetic beads (**Figure** **4**a). ATRA‐LNP in phosphate‐buffered saline (PBS) or free ATRA in DMSO were incubated with T cells during stimulation. The use of ATRA‐LNP allowed us to take advantage of the enhanced water solubility of encapsulated ATRA compared to free ATRA, thus avoiding the use of a toxic organic solvent. The expressions of CCR9 and α4β7 on activated T cells were then analyzed by flow cytometry. The results showed that ATRA‐LNP and free ATRA significantly increased the expressions of CCR9 and α4β7 on CD8 and CD4 T cells compared to the PBS group (Figure 4b–j; Figure S4, Supporting Information). ATRA‐LNP treatment increased ≈59% of CD3^+^CD8^+^CCR9^+^, 14% of CD3^+^CD8^+^α4β7^+^, and 71% of CD3^+^CD8^+^α4β7^+^CCR9^+^ T cell populations compared to free ATRA treatment, indicating a higher efficacy on inducing gut‐homing receptors of CD8^+^ T cells. The increased efficacy of ATRA‐LNP could be attributed to the enhanced water solubility of ATRA compared to free ATRA, which might precipitate after being added to the aqueous cell culture medium. Collectively, these findings demonstrated that ATRA‐LNP significantly increases the water solubility of ATRA and enhances its efficacy in inducing gut‐homing receptors of T cells.

### 2.4 ATRA‐LNP Activates Systemic and Mucosal Immune Responses In Vivo

**Figure 5:**
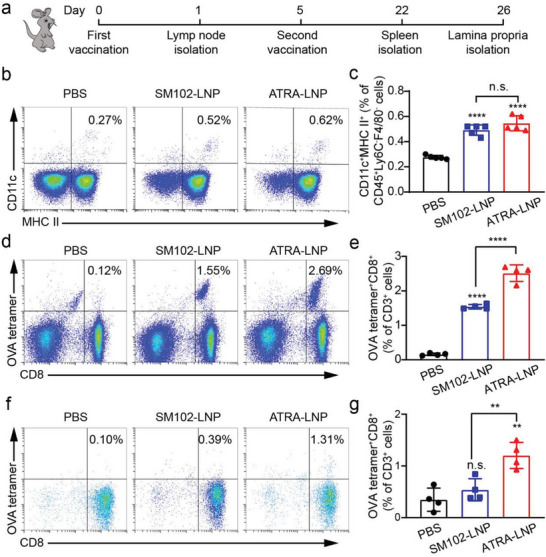
a) Schematic of vaccination regimen on C57BL/6 mice. Mice were vaccinated through intramuscular injections at days 0 and 5. The draining lymph nodes, spleens, and intestine lamina propria were isolated for flow cytometry analysis on days 1, 22, and 26, respectively. Representative flow cytometry plots and quantification of activated DCs b and c) in the draining lymph nodes (n = 5 biologically independent samples), OVA tetramer^+^CD8^+^ T cells d and e) in the spleen, and OVA tetramer^+^CD8^+^ T cells (f and g) in the intestine lamina propria (n = 4 biologically independent samples) after different treatments. Data are shown as mean ± SD. Statistical analysis was performed using one‐way ANOVA and Tukey's multiple comparisons tests: ^**^ *p* <0.01, ^****^ *p* <0.0001, n.s. represents not statistically significant.

To evaluate the ability of ATRA‐LNP to activate antigen‐presenting cells and induce T cell homing to the gut in vivo, we prepared LNPs encapsulating ovalbumin‐encoded mRNA (mOVA) as a model antigen. Female C57BL/6 mice were vaccinated with SM102‐LNP or ATRA‐LNP containing 10 µg of mOVA per dose via intramuscular injections at days 0 and 5. On day 1, we isolated the draining lymph node near the injection site to evaluate the activation of DCs (**Figure** **5**a). Both SM102‐LNP and ATRA‐LNP treatments increased the size and weight of draining lymph nodes (Figure S5, Supporting Information). Flow cytometry measurements showed that SM102‐LNP and ATRA‐LNP significantly increased the populations of CD11c^+^MHCII^+^CD45^+^Ly6C^−^F4/80^−^ DCs (Figure 5b,c), CD11b^+^Ly6C^high^CD45^+^ inflammatory monocytes and CD11b^+^F4/80^+^CD45^+^ macrophages compared to the PBS group (Figure S6, Supporting Information), indicating effective activation of DCs and the innate immune responses. ATRA‐LNP showed similar DC activation efficacy to SM102‐LNP, suggesting that ATRA did not contribute to DC activation. To evaluate the activation of systemic immune responses, we isolated splenocytes at day 22 post‐first vaccination and analyzed the population of antigen‐specific cytotoxic T cells. The amount of OVA tetramer^+^CD8^+^CD3^+^ T cells among splenocytes in the ATRA‐LNP treated groups was ≈1.6 times and 16 times as that of the SM102‐LNP and PBS treatments, respectively, suggesting effective activation of systemic T‐cell response (Figure 5d,e). The enhanced T‐cell response for ATRA‐LNP in the spleen was probably because of its higher mRNA expression.

The goal of incorporating ATRA into LNP was to induce T cell homing to the guts. We then isolated the lamina propria of the intestine and examined the population of antigen‐specific CD8^+^ T cells. As shown in Figure 5f,g, SM102‐LNP without ATRA exhibited a nearly identical population of OVA tetramer^+^CD8^+^CD3^+^ T cells as the PBS treatment, suggesting negligible homing of activated T cells to the intestine. In contrast, ATRA‐LNP significantly increased the amount of OVA tetramer^+^CD8^+^CD3^+^ T cells compared to SM102‐LNP, suggesting that incorporated ATRA imprinted activated T cells with gut‐homing tropism. Overall, our results showed that ATRA‐LNP activated DCs in the draining lymph node, elicited a strong systemic cellular immune response, and increased the accumulation of antigen‐specific, cytotoxic T cells in the gut.

### 2.5 ATRA‐LNP Inhibits The Growth of Orthotopic Colorectal Tumors In Mice

**Figure 6:**
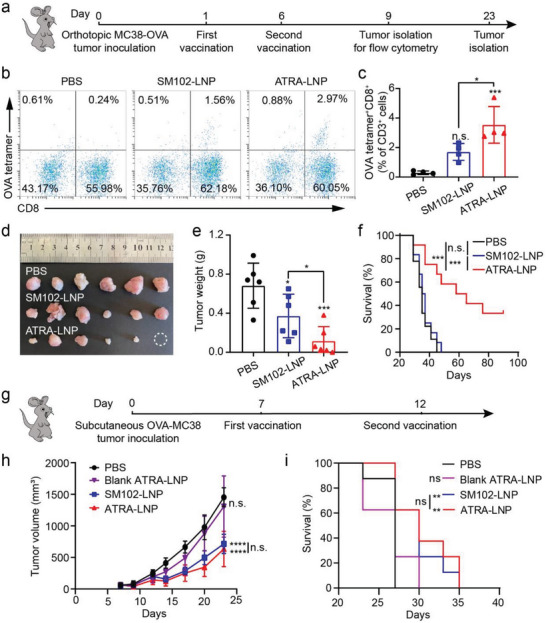
a) Treatment scheme of an orthotopic MC38‐OVA colorectal tumor model using mOVA‐loaded LNPs. b and c) Representative flow cytometry plots and quantification of OVA tetramer^+^CD8^+^ T cells in the tumors after different treatments (n = 4 biologically independent samples). Statistical significance was calculated by one‐way ANOVA and Tukey's multiple comparisons test. d and e) Photographs and weights of isolated tumors (n = 6 biologically independent samples). Statistical significance was calculated by unpaired two‐tailed Student's *t*‐test. f) Kaplan–Meier survival curves of mice after different treatments (n = 9 – 12 biologically independent samples per group). g) Treatment scheme of a subcutaneous MC38‐OVA colorectal tumor model. h and i) Average tumor growth and Kaplan–Meier survival curves of tumor‐bearing mice after different treatments (n = 8 biologically independent samples). Data are shown as mean ± SD. Statistical significance was calculated by two‐way ANOVA and Tukey's multiple comparisons test: ^*^ *p* < 0.05, ^**^ *p* <0.01, ^***^ *p* <0.001, ^****^ *p* <0.0001, n.s. represents not statistically significant.

The ability to induce the trafficking of cytotoxic T cells into the gut makes ATRA‐LNP a promising therapeutic vaccine to treat colorectal cancer. We next evaluated the antitumor efficacy of ATRA‐LNP as a therapeutic vaccine in an orthotopic colorectal cancer model in mice. To establish the cancer model, we used a mouse colorectal cancer cell line MC38 that was stably transfected to express OVA. We then transplanted a small piece of subcutaneous MC38‐OVA tumor (3–4 mg) onto the cecum following a previously published method.^[^ ^40^ ^]^ The tumor‐bearing mice were vaccinated with SM102‐LNP or ATRA‐LNP via intramuscular injections at days 1 and 6 post‐tumor inoculation (**Figure** **6**a). Each dose of the vaccine contains 10 µg of mOVA and 70 µg of ATRA (if applicable) per mouse. On day 3 post the second vaccination, we isolated the orthotopic tumors and analyzed the number of tumor‐infiltrating cytotoxic T cells, which is a key parameter of evaluating antitumor immune responses. As shown in Figure 6b, SM102‐LNP induced a higher population of OVA tetramer^+^CD8^+^CD3^+^ T cells compared to PBS treatment, suggesting that the mRNA vaccine was in general effective in inducing an anti‐tumor immune response. ATRA‐LNP treatment induced ≈2.1‐ and 12.2‐times OVA tetramer^+^CD8^+^CD3^+^ T cells as the SM102‐LNP and PBS treatments, respectively (Figure 6c; Figure S7, Supporting Information). These results indicated that ATRA in the LNP imprints activated T cells with gut tropism and increased the infiltration of antigen‐specific, cytotoxic T cells into the tumor microenvironment, which could contribute to better therapeutic effects against tumors. Indeed, ATRA‐LNP treatment significantly reduced tumor growth compared with SM102‐LNP and PBS treatments. At day 23 post‐tumor inoculation, the tumor weight of ATRA‐LNP treated mice decreased to 16.9% and 30.9% compared with PBS and SM102‐LNP treatments, respectively (Figure 6d,e). ATRA‐LNP also significantly extended the survival of tumor‐bearing mice (Figure 6f). In contrast, SM102‐LNP showed limited efficacy in inhibiting tumor growth and did not extend animal survival compared to the PBS group. These data demonstrated that ATRA‐LNP augments the antitumor immune responses and effectively inhibits the growth of orthotopic colon tumors.

To further demonstrate the essential role of ATRA in LNP on inducing the mucosal antitumor immune response for inhibiting orthotopic tumor growth, we evaluated the therapeutic efficacy of ATRA‐LNP or SM102‐LNP encapsulating mOVA using a subcutaneous MC38‐OVA model in mice (Figure 6g). As shown in Figure 6h,i, SM102‐LNP, which exhibited nearly no therapeutic efficacy on orthotopic MC38‐OVA tumors, greatly inhibited subcutaneous MC38‐OVA tumor growth and extended animal survival, indicating that mRNA cancer vaccines encapsulated in traditional LNP (e.g., SM102‐LNP) are effective on treating subcutaneous tumors but have limited efficacy on treating orthotopic colorectal tumors. ATRA‐LNP showed similar therapeutic efficacy compared to SM102‐LNP. Blank ATRA‐LNP (without mOVA) treatment showed comparable tumor growth and survival compared to PBS treatment, suggesting that ATRA itself did not directly inhibit tumor growth. Collectively, these data in turn demonstrated that the superior therapeutic efficacy on orthotopic colon tumors of ATRA‐LNP over SM102‐LNP is due to ATRA‐induced anti‐tumor immune responses in the gut.

**Figure 7:**
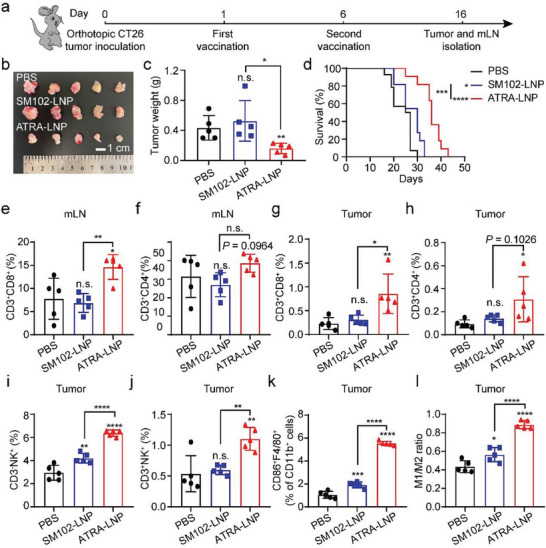
a) Treatment scheme of an orthotopic CT26 colorectal tumor model using mGP70‐loaded LNPs. (b and c) Photographs and weights of isolated tumors (n = 5 biological independent samples). Statistical significance was calculated by unpaired two‐tailed Student's *t*‐test. d) Kaplan–Meier survival curves of mice after different treatments (n = 11–14 biologically independent samples). The percentages of CD3^+^CD8^+^ and CD3^+^CD4^+^ T cells in mLN e and f) and tumor g and h) of treated mice. The population of CD3^−^NK1.1^+^ i), CD3^+^NK1.1^+^ j), and CD86^+^F4/80^+^ cells among tumor cells. k) M1/M2 ratio in the tumor microenvironment (n = 5 biological independent samples for all flow cytometry analysis). Data are shown as mean ± SD. Statistical analysis was calculated using one‐way ANOVA and Tukey's post‐hoc test. ^*^ *p* < 0.05, ^**^ *p* <0.01, ^***^ *p* <0.001, ^****^ *p* <0.0001, n.s. represents not statistically significant.

To assess the efficacy of ATRA‐LNP in delivering a native tumor antigen instead of OVA, we designed an mRNA encoding the envelope glycoprotein 70 (mGP70), which is universally expressed by a variety of murine cancer cell lines including CT26 (a mouse colon carcinoma cell line). The overexpression of gp70 in CT26 is confirmed by quantitative real‐time polymerase chain reaction (Figure S8, Supporting Information). We first established orthotopic CT26 colon tumors by transplanting a small piece of subcutaneous CT26 tumor (3–4 mg) onto the cecum of BALB/c mice. SM102‐LNP or ATRA‐LNP encapsulating mGP70 (10 µg per mouse per dose) were intramuscularly injected into mice on days 1 and 6 post‐tumor inoculation (**Figure** **7**a). As shown in Figure 7b,d, ATRA‐LNP inhibited tumor growth and prolonged animal survival compared to SM102‐LNP or PBS. SM102‐LNP slightly prolonged animal survival but did not show efficacy in inhibiting primary tumor growth. No metastasis to the liver or any major organs was observed for all groups (Figure S9, Supporting Information). Flow cytometry analysis showed that ATRA‐LNP enhanced CD8 and CD4 T cell infiltrations in the tumor and mesenteric lymph nodes (Figure 7e–h; Figures S10 and S11, Supporting Information). These data are consistent with the results obtained in the orthotopic MC38‐OVA model. We further analyzed the population of macrophages, natural killer cells (NKs), and exhausted T cells in tumors. As shown in Figure 7i–k and Figure S12 (Supporting Information), ATRA‐LNP greatly increased the population of NKs (NK1.1^+^CD3^−^), NK T cells (NK1.1^+^CD3^+^), and M1 macrophages compared to SM102‐LNP and PBS treatments, suggesting that ATRA‐LNP induced an innate inflammatory niche with the potential to prime adaptive immunity. ATRA‐LNP also increased the M1 to M2 macrophage ratios, indicating a less immunosuppressive tumor microenvironment (Figure 7l). We next evaluated the exhaustion of CD8^+^ T cells in the tumor. Both SM102‐LNP and ATRA‐LNP greatly reduced the expression of PD1^+^ among CD8^+^ T cells, indicating the function of CD8^+^ T cells in the tumor microenvironment was partially restored (Figure S13, Supporting Information). Recent studies showed that low PD1 expression among CD8+ T cells may lead to poor response to anti‐PD1 blockade therapies.^[^ ^41^, ^42^, ^43^ ^]^ Therefore, we did not further explore the combination of ATRA‐LNP and immune checkpoint blockade therapy.

### 2.6 Toxicity Analysis of ATRA‐LNP

ATRA is an active metabolite of vitamin A and has been clinically approved to treat acne and acute promyelocytic leukemia. The dosage of ATRA to treat leukemia is 45 mg m^−2^ day^−1^ in two divided doses,^[^ ^44^ ^]^ which is significantly higher than the dose used in the current study. Therefore, adding ATRA to clinically approved LNP is unlikely to induce additional side effects or toxicity. To assess the safety of ATRA‐LNP, we monitored the body weight of mice after vaccination. We did not observe significant changes in body weights for all groups throughout the treatment period (Figure S14, Supporting Information). Additional hematoxylin and eosin (H&E) staining of major organs including the heart, liver, spleen, lung, and kidney, showed no obvious change in morphology (Figure S15, Supporting Information), suggesting that ATRA‐LNP did not induce any significant adverse effects in the mice. However, further studies are needed to comprehensively evaluate the safety of ATRA‐LNP in larger animals and eventually in clinical trials.

## 3 Conclusion

In summary, we developed a mucosal mRNA cancer vaccine that can activate anti‐tumor immune responses on the mucosal surface of the gut through intramuscular injection. The incorporation of ATRA into LNP not only enhanced its mRNA delivery efficiency and systemic T‐cell responses in the spleen but also imprinted activated T cells with gut tropism, which significantly increased the infiltration of cytotoxic T cells into orthotopic colorectal tumors, resulting in augmented therapeutic efficacy. These results suggest that ATRA could be used as an adjuvant for the mRNA vaccine to boost mucosal immune responses in the gut. LNP also serves as a delivery vehicle of ATRA to improve its water solubility, therefore, avoiding the use of a toxic solvent to dissolve and administer ATRA. In terms of translational potential, all the components in ATRA‐LNP are clinically approved. Our results suggest that adding a small amount of ATRA as an adjuvant into LNP did not cause additional side effects or toxicity in animal studies. The preparation process of ATRA‐LNP is the same as the approved COVID‐19 mRNA vaccines. Therefore, we do not anticipate significant challenges in scale‐up productions. Overall, our approach of co‐delivering ATRA and mRNA cancer vaccines through LNP represents a simple and promising strategy to improve the therapeutic efficacy of current mRNA cancer vaccines against colorectal cancer.

## 4 Experimental Section

### Materials

Chemicals and reagents were purchased from Sigma–Aldrich, Thermo Scientific, and Beijing Innochem Science & Technology unless noted specifically. SM102 was purchased from Sinopeg. DSPC and DMG‐PEG2000 were purchased from Avanti Polar Lipids. mRNAs were purchased from ApexBio or TriLink Biotechnologies. D‐luciferin potassium salt was purchased from Bide Pharm.

### Preparation and Characterization of LNPs

LNPs were synthesized by mixing the lipids and mLuc in a microfluidic chip. Briefly, SM102, DSPC, cholesterol, and DMG‐PEG2000 at a molar ratio of 50:10:38.5:1.5, respectively, were dissolved in the ethanol phase. For ATRA LNPs, different concentrations of ATRA in ethanol were mixed with lipid solutions. mLuc was dissolved in 50 mm citrate buffer (pH 4) to obtain an aqueous phase. The ratio of nitrogen on ionizable lipids to the phosphate of mRNA is 5.67:1 or 15:1. The ethanol phase and aqueous phase were mixed with the volume ratio of 3:1 using syringe pumps (Harvard Apparatus). The mixture was dialyzed (MWCO = 12k–14 kDa, Biorigin) against PBS at 4 °C for over 4 h. The hydrodynamic diameter, polydispersity index, and zeta‐potentials of LNPs were measured at 25 °C by Zetasizer Nano ZSP (Malvern Instruments). The encapsulation efficiency was measured using Quant‐iT RiboGreen RNA assay (Thermo Fisher Scientific) according to the manufacturer's instructions. Cryo‐EM image of ATRA‐LNP was acquired using a Themis 300 electron microscope. The preparation of LNPs was repeated over five times.

### Quantification of ATRA by HPLC

Standard ATRA solutions were prepared at concentrations of 1, 6.25, 12.5, 25, 50, 100, and 200 µg mL^−1^ in ethanol. ATRA solutions were then subjected to HPLC analysis that was performed on a Waters Breeze HPLC system equipped with a SunFire C18 column (5 µm, 4.6 × 150 mm) and a photodiode array detector. The mobile phases were acetonitrile containing 0.1% of trifluoroacetic acid (TFA) and triethylamine acetate buffer (0.1 m) containing 0.1% of TFA. A standard curve between the concentration and area under the curve of ATRA was then established. To quantify ATRA in ATRA‐LNP, 2 µL of ATRA‐LNP in PBS was dissolved in 98 µL of ethanol and analyzed by HPLC. The concentration of ATRA was then calculated according to the standard curve.

### In Vitro mRNA Delivery

The Mouse DC2.4 cells were cultured in Roswell Park Memorial Institute (RPMI) 1640 medium supplemented with 10% fetal bovine serum (FBS), 100 U mL^−1^ of penicillin, and 100 µg mL^−1^ of streptomycin at 37 °C in 5% CO_2_. To evaluate the mRNA delivery efficiency in vitro, DC2.4 cells were seeded at a density of 2 × 10^4^ cells per well in a Corning Costar 96‐well plate. After incubation at 37 °C overnight, LNPs containing 100 ng of mRNA were added to each well. After incubation at 37 °C for 24 h, 100 µL of the medium was removed before adding One‐lite Luciferase Assay substrate in 100 µL of lysis buffer to each well. The luminescence was then read by a BioTek Synergy H1 multimode microplate reader (Agilent). The in vitro mRNA delivery was repeated at least three times with four technical replicates per group.

### Cellular Uptake of LNPs

To evaluate the cellular uptake, DC2.4 cells were seeded at a density of 2 × 10^4^ cells per well in a 96‐well plate and incubated overnight. Subsequently, the cells were incubated with LNPs containing 100 ng FAM‐A_20_ or free FAM‐A_20_ for 3 h. The cells were then washed with PBS and collected for flow cytometry analysis using a CytoFLEX flow cytometer (Beckman Coulter).

### Lipid Fusion Assay

Endosomal vesicles were formulated with a lipid composition of 42% 1,2‐dioleoyl‐sn‐glycero‐3‐phosphoethanolamine (DOPE), 17% 1,2‐dioleoyl‐sn‐gylcero‐3‐phosphocholine (DOPC), 13% bis(monooleoyglycero)phosphate(S,R Isomer) (ammonium salt) (LBPA), and 28% cholesterol, and included two dye‐labeled lipids, NBD‐PE (λ_EX_/λ_EM_ = 463 nm/536 nm) and Rhod‐PE (λ_EX_/λ_EM_ = 570 nm/590 nm), for fluorescence detection. To prepare the vesicles, the ethanol phase containing the lipid mixture was combined with PBS at pH 5.5. This solution was then subjected to dialysis (using a membrane with MWCO of 12 000–14 000 Da, Biorigin) against PBS (pH 5.5) at 4 °C for over 4 h. The successful synthesis of these vesicles was confirmed using dynamic light scattering. Lipid nanoparticles (LNPs) encapsulating mRNA were administered at a series of concentrations. After incubating at room temperature, fluorescence measurements (F) were conducted on a microplate reader at Ex/Em = 463/590 nm. The lipid fusion (%) was calculated as (1‐F/F_max_) × 100%. F_max_ was the initial value of fluorescence measurements.

### RT‐qPCR

Total RNAs of CT26 and DC2.3 cells were extracted using Trizol reagent (Beyotime) according to the manufacturer's instructions. cDNA was then synthesized using HiScript III 1^st^ Strand cDNA Synthesis Kit (+gDNA wiper) (Vazyme). Then cDNA and primers were mixed with Taq Pro Universal SYBR qPCR Master Mix (Vazyme). RT–qPCR was performed on a real‐time fluorescence quantitative PCR instrument (RocGene, Archimed X4). The primer sequences were gp70 forward (5′‐ctggactcactccctgtatc‐3′), gp70 reverse (5′‐caaattggtggtaaacataactagggg‐3′), GAPDH forward (5′‐tgcaccaccaactgtttagc‐3′), and GAPDH reverse (5′‐ggcatggactgtggtcatgag‐3′).

### Animals

All animal procedures were performed according to a protocol approved by the Peking University Institutional Animal Care and Use Committee (No. LA2021284 and No. LA2023163). Female C57BL/6 or BALB/c mice were purchased from SPF (Beijing) Biotechnology or Peking University Health Science Center Department of Laboratory Animal Science.

### In Vivo mRNA Delivery

To determine the in vivo delivery efficiency of LNPs, C57BL/6 mice were administered intramuscularly with 30 µL of LNP containing 1 µg of mLuc. The mice were then injected intraperitoneally with 150 µL of D‐luciferin potassium salt in PBS (20 mg mL^−1^) before imaging. After 10 min, the mice were imaged by an in vivo imaging system (IVIS, PerkinElmer). The luminescence was quantified using the Living Image software (PerkinElmer). The in vivo mRNA delivery was performed twice with four biologically independent replicates per group.

### Quantification of CCR9 and α4β7 on T Cell

Mouse T cells were isolated from the spleen using EasySep Mouse CD8^+^ T Cell Isolation Kit (Stem Cell Technology) according to the manufacturer's instructions. Isolated CD8^+^ T cells were seeded into 96‐well plates at a density of 1 × 10^5^ cells per well in 100 µL of RPMI 1640 medium supplemented with 10% of FBS, 1% of penicillin/streptomycin, 1% of non‐essential amino acids, 1% of sodium pyruvate, 2‐mercaptoethanol, and 100 U mL^−1^ of Interleukin‐2 (Gibco). LNPs in PBS or free ATRA in dimethyl sulfoxide (DMSO) were added to each well at the same concentration of ATRA (1 nm). CD8^+^ T cells were then incubated with a Mouse T Cell Activation/Expansion Kit (Miltenyi). After 5 days of incubation, CD8^+^ T cells were collected, incubated with anti‐CD16/32 antibodies first, and then stained with mouse anti‐CD3‐fluorescein isothiocyanate (FITC) (BioLegend, 100204), anti‐CCR9‐ phycoerythrin (PE) (BioLegend, 129708), and anti‐α4β7‐allophycocyanin (APC) (BioLegend, 120607) antibodies for 30 min at 4 °C. CD8^+^ T cells were then washed with PBS for two times and analyzed using a CytoFLEX flow cytometer. To measure the expression of CCR9 and α4β7 on CD4 T cells, Spleen lymphocytes were isolated by Ficoll‐Paque PREMIUM 1.084 sterile solution (Cytiva). Isolated spleen lymphocytes were seeded into 48‐well plates at a density of 5 × 10^5^ cells per well in 500 µL of RPMI 1640 supplemented with 10% of FBS, 1% of penicillin/streptomycin, 1% of non‐essential amino acids, 1% of sodium pyruvate, 2‐mercaptoethanol, and 100 U mL^−1^ of Interleukin‐2. LNPs in PBS or free ATRA in DMSO were added to each well at the same concentration of ATRA (10 nm). Spleen lymphocytes were then incubated with a Mouse T Cell Activation/Expansion Kit. After 4 days of incubation, Spleen lymphocytes were collected, incubated with anti‐CD16/32 antibodies first, and then stained with mouse anti‐CD3‐FITC (BioLegend, 100204), anti‐CD4‐peridinin‐chlorophyll‐protein (PerCP) (Biolegend, 100537), anti‐CCR9‐ phycoerythrin (PE) (BioLegend, 129708), and anti‐α4β7‐allophycocyanin (APC) (Biolegend, 120607) antibodies for 30 min at 4 °C. Spleen lymphocytes were then washed with PBS for two times and analyzed using a CytoFLEX flow cytometer. Quantification of CCR9 and α4β7 on T cells was performed twice with three to four technical replicates per group.

### Immune Responses of LNPs In Vivo

Mice were vaccinated intramuscularly with 100 µL of PBS, SM102‐LNP (10 µg of mRNA), or ATRA‐LNP (10 µg of mRNA and 70 µg of ATRA) at days 0 and 5. To evaluate DC activation in the draining lymph node. Mice were sacrificed at 24 h post‐first vaccination. The inguinal lymph nodes on the same side of the injection were isolated and digested into single‐cell suspensions for flow cytometry. After incubation with anti‐CD16/32 antibodies, the cell suspensions were stained with anti‐CD45‐PerCP (BioLegend, 103130), anti‐CD11b‐APC (BioLegend, 101211), anti‐F4/80‐PE (BioLegend, 123110), anti‐Ly6C‐PE/Cyanine7 (BioLegend, 128017), anti‐CD11c‐FITC (BioLegend, 117305), and anti‐I‐A/I‐E (MHC II)‐Alexa Fluor 700 monoclonal (Biolegend, 107622) antibodies and analyzed by flow cytometry. DC activation in the draining lymph node was performed twice with five biologically independent replicates per group. To analyze systemic T‐cell responses, the spleens of mice were isolated at day 22 post‐first vaccination and digested into single‐cell suspensions. After incubation with anti‐CD16/32 antibodies, the splenocytes were stained with T‐Select H‐2Kb OVA tetramer‐SIINFEKL‐APC (MBL, TS‐5001‐2C), anti‐CD8‐FITC (MBL, K0227‐4), and anti‐CD3‐PE (eBioscience, 12‐0032‐82) antibodies before flow cytometric analysis.

The isolation of the lamina propria lymphocytes (LPL) was performed according to a previously published protocol.^[^ ^45^ ^]^ The small intestines of mice were isolated and immersed in Hank's Balanced Salt Solution (HBSS, Solarbio) supplemented with 5% FBS. The surrounding fat tissues of the intestine, the Peyer's patches, and feces were removed. The intestine was cut into 1 cm pieces, which were then placed into pre‐heated HBSS containing 2% FBS, 2.5 mm ethylene diamine tetraacetic acid (EDTA), and 1 mm dithiothreitol (DTT) and shaken for 20 min at 37 °C. The mixture was filtered by a 70 µm cell strainer. The remained intestine tissue was minced after being washed with PBS. The minced intestine was transferred to complete RPMI 1640 containing 1 mg mL^−1^ collagenase IV and 0.2 mg mL^−1^ DNase I and shaken for 20 min. The mixture was passed through a 70 µm cell strainer. The cell suspension was centrifuged at 300 g for 5 min. The cell pellet was then suspended in Ficoll‐Paque PLUS density gradient media (GE Healthcare) before adding 1 mL of culture media. The cells were then centrifuged at 800 g for 30 min. The top layer of cells on gradient media was collected. After incubation with anti‐CD16/32 antibodies, cells were stained with APC H‐2Kb/SIINFEKL tetramer (MBL, TS‐5001‐2C), anti‐CD8‐FITC (MBL, K0227‐4), and anti‐CD3‐PE (eBioscience, 12‐0032‐82) antibodies before flow cytometric analysis.

### Tumor Inhibition Against Orthotopic Mouse Models of Colorectal Cancer

A mouse colon cancer cell line MC38‐OVA was purchased from Tongpai Biotechnology (Shanghai). MC38‐OVA cells were cultured in Dulbecco's Modified Eagle Medium (DMEM) supplemented with 10% fetal bovine serum, 100 U mL^−1^ of penicillin, and 100 µg mL^−1^ of streptomycin. Mice were injected subcutaneously with 1.0 × 10^6^ of MC38‐OVA cells in 200 µL of PBS into the right flank. Mice were euthanized when the tumor grew to ≈500 mm^3^. The tumors were isolated and divided into 3 to 4 mg pieces. The tumor tissue was then transplanted to the cecum of healthy mice to establish orthotopic colorectal tumors in mice. The specific steps of transplantation were according to a previously published protocol. The mice were intramuscularly vaccinated with PBS, SM102‐LNP, or ATRA‐LNP on days 1 and 6 post‐tumor inoculations. The doses of mOVA and ATRA were 10 and 70 µg, respectively. The body weight of mice was measured every 3 days post‐tumor inoculation. The tumors were isolated at day 9 post‐tumor inoculation to evaluate the infiltration of T cells. The single‐cell suspension of tumors was prepared, blocked with anti‐CD16/32 antibodies, and stained with APC H‐2Kb/SIINFEKL tetramer (MBL, TS‐5001‐2C), anti‐CD8‐FITC (MBL, K0227‐4), and anti‐CD3‐PE (eBioscience, 12‐0032‐82) antibodies before flow cytometric analysis. To evaluate the tumor inhibition efficacy, the tumors on the cecum and the major organs including the heart, liver, spleen, lung, and kidney were isolated at day 23 post‐tumor inoculation. The tumors of each group were imaged and weighed. The major organs were fixed in 4% paraformaldehyde, embedded in paraffin, and sectioned into 5 µm slices for H&E staining.

The orthotopic CT26 tumor model was established using the same methods as the orthotopic MC38‐OVA tumor model. PBS, SM102‐LNP, or ATRA‐LNP were injected intramuscularly on days 1 and 6 post‐tumor inoculation. The dose of gp70 mRNA and ATRA‐LNP were 10 and 70 µg, respectively. Following tumor inoculation, the body weight of the mice was monitored and recorded at three to four‐day intervals. The mesenteric lymph nodes and tumors were isolated on day 16 post‐tumor inoculation to evaluate the changes in immune cells. The tumors on the cecum of each group were imaged and weighed. The single‐cell suspension of mesenteric lymph nodes was prepared, blocked with anti‐CD16/32 antibodies, and stained with anti‐CD3‐BV421 (BioLegend, 100227), anti‐CD4‐PerCP (BioLegend, 100537), and anti‐CD8‐PE (Biolegend, 100708) antibodies before flow cytometric analysis. The single‐cell suspension of tumors was prepared, blocked with anti‐CD16/32 antibodies, and staining with anti‐NK1.1‐PE (BD Biosciences, 557391), anti‐CD3‐FITC (BioLegend, 100204), anti‐CD11b‐APC (BioLegend, 101211), anti‐F4/80‐Brilliant Violet 510 (BV510) (BioLegend, 123135), anti‐CD86‐BV421 (BioLegend, 105031), and anti‐CD206‐PerCP/Cy5.5 (BioLegend, 141715), or anti‐CD3‐FITC (BioLegend, 100204), anti‐CD4‐PerCP (BioLegend, 100537), anti‐PD1‐PE(BioLegend, 135205), and anti‐CD8‐APC (eBioscience, 17‐0081‐82) antibodies before flow cytometric analysis. MC38‐OVA orthotopic tumor inhibition was performed three times with six to nine biologically independent replicates per group. The flow cytometric analysis of T cells in the tumor was repeated twice with four biologically independent replicates per group on MC38‐OVA orthotopic tumor or CT26 orthotopic tumor.

### Statistical Analysis

All statistical analyses were performed using the GraphPad Prism software package. Technical or biological replicates were used in all experiments unless otherwise stated. Data were presented as means ± SD. The specific statistical methods were indicated in the figure legends. GraphPad Prism was used for statistical analysis. A two‐tailed Student's *t*‐test or a one‐way analysis of variance (ANOVA) with a Tukey's post hoc test was adopted for comparing two groups or more than two groups, respectively. Data were significantly different if *p* < 0.05 (^*^ *p* < 0.05, ^**^ *p* < 0.01, ^***^ *p* < 0.001, ^****^ *p* < 0.0001).

## Conflict of Interest

The authors declare no conflict of interest.

## Supporting information

Supporting Information

## Data Availability

The data that support the findings of this study are available from the corresponding author upon reasonable request.
