# Supplementary material for: All‐Trans‐Retinoic Acid‐Adjuvanted mRNA Vaccine Induces Mucosal Anti‐Tumor Immune Responses for Treating Colorectal Cancer
Source: Adv Sci (Weinh). 2024 Mar 25;11(22):2309770. doi: 10.1002/advs.202309770 (PMC11165559; doi:10.1002/advs.202309770)
Supplement: Supplementary file 1 — Supporting Information [file ADVS-11-2309770-s001.pdf]

ADVANCED SCIENCE
Open Access

Supporting Information

for *Adv. Sci.*, DOI 10.1002/advs.202309770

All-*Trans*-Retinoic Acid-Adjuvanted mRNA Vaccine Induces Mucosal Anti-Tumor Immune Responses for Treating Colorectal Cancer

Wei Li, Yijia Li, Jingjiao Li, Junli Meng, Ziqiong Jiang, Chen Yang, Yixing Wen, Shuai Liu, Xingdi Cheng, Shiwei Mi, Yuanyuan zhao, Lei Miao and Xueguang Lu**

Supporting Information

All-*trans*-retinoic acid-adjuvanted mRNA vaccine induces mucosal anti-tumor immune responses for treating colorectal cancer

Wei Li[1,2,#], Yijia Li[3,4,#], Jingjiao Li[1,2], Junli Meng[1,2], Ziqiong Jiang[3,4], Chen Yang[1,2], Yixing Wen[1,2], Shuai Liu[1], Xingdi Cheng[1], Shiwei Mi[1,2], Yuanyuan zhao[1,2], Lei Miao[3,4*] and Xueguang Lu[1,2*]

1. Beijing National Laboratory for Molecular Sciences, CAS Key Laboratory of Colloid, Interface and Chemical Thermodynamics, Institute of Chemistry, Chinese Academy of Sciences, Beijing 100190, China

2. University of Chinese Academy of Sciences, Beijing 100049, China

3. State Key Laboratory of Natural and Biomimetic Drugs, School of Pharmaceutical Sciences, Peking University, Beijing 100191, China

4 Beijing Key Laboratory of Molecular Pharmaceutics and New Drug Delivery System, School of Pharmaceutical Sciences, Peking University, Beijing 100191, China

# These authors contributed equally.

*Email: lmiao_pharm@bjmu.edu.cn (L.M.); xueguang@iccas.ac.cn (X.L.)

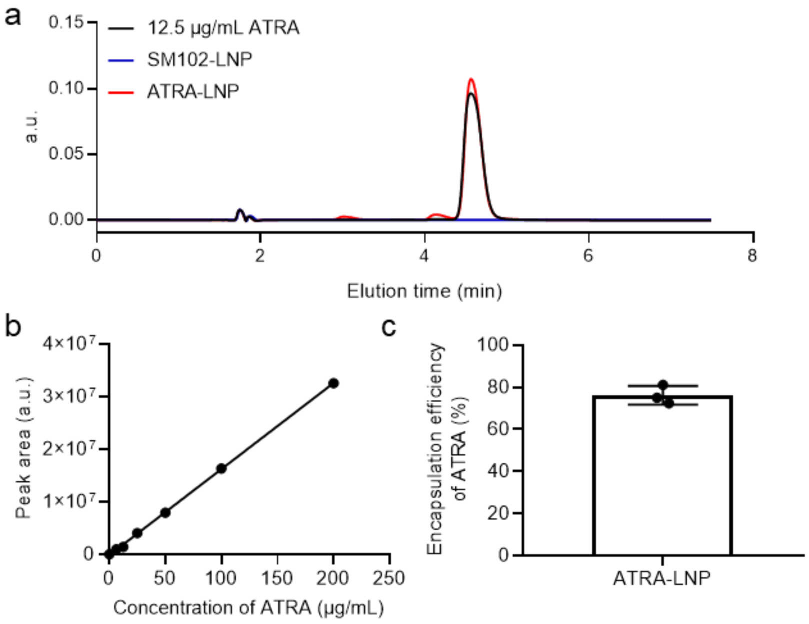

Figure S1. (a) HPLC chromatograms of free ATRA, SM102-LNP, and ATRA-LNP (N/P=15) at 340 nm. (b) HPLC standard curve of free ATRA. (c) The encapsulation efficiency of ATRA in ATRA-LNP (n=3). Data are shown as mean ± SD.

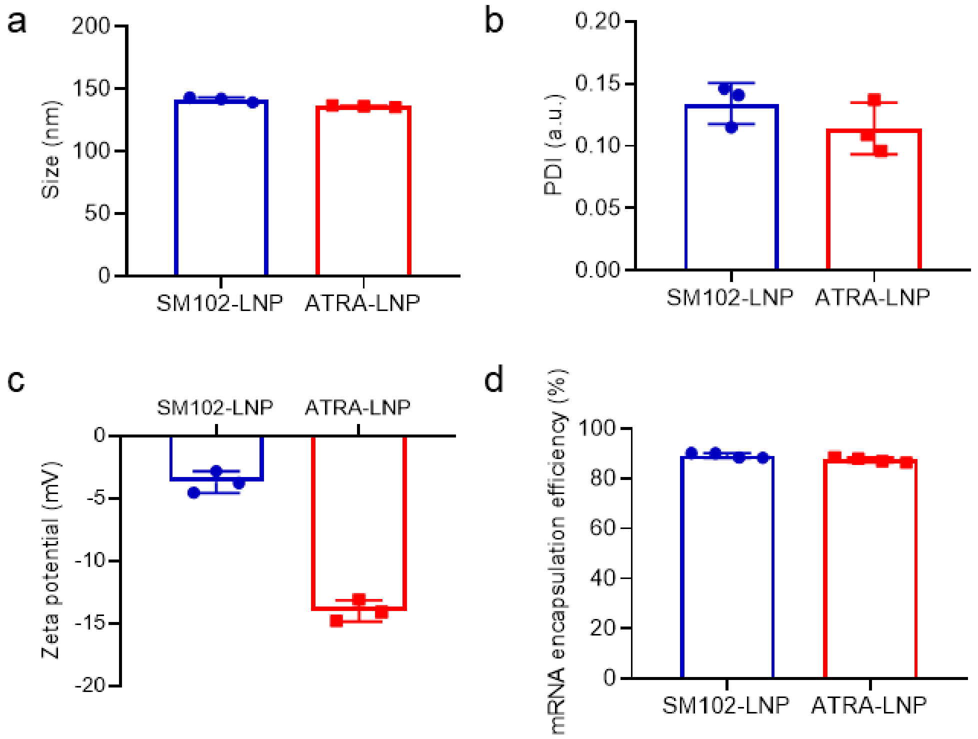

Figure S2. Characterizations of LNPs (N/P=15). The hydrodynamic diameter (a), PDI (b), zeta potential (c), and mRNA encapsulation efficiency (d) of SM102-LNP and ATRA-LNP. n = 3 or 4 technical replicates. Data are shown as mean ± SD.

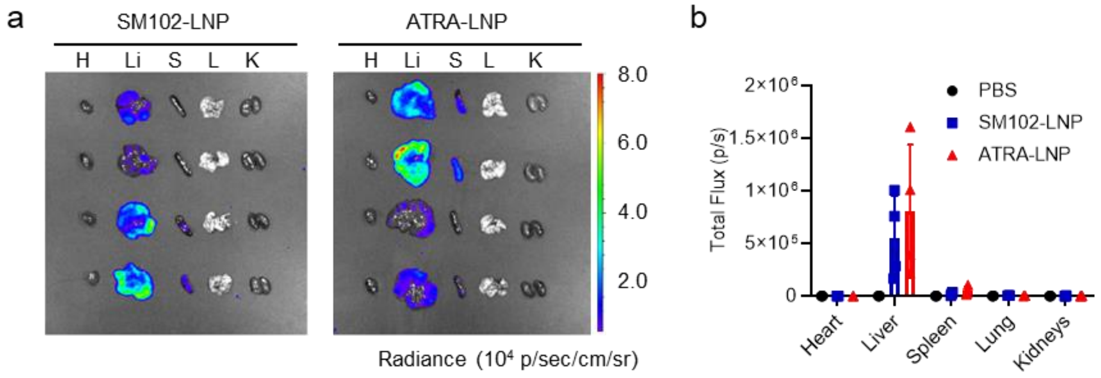

Figure S3. Ex vivo image of mice organs. (a)IVIS images of isolated organs (H: heart; Li: Liver; S: spleen; L: lung; K: kidneys) of mice at 24 h post intramuscular injection of SM102-LNP or ATRA-LNP (1 μg of mLuc per mouse). Each vertical column of organs represents four replicates. (b) Quantification of bioluminescence signals of isolated organs (n = 4). Data are shown as mean ± SD.

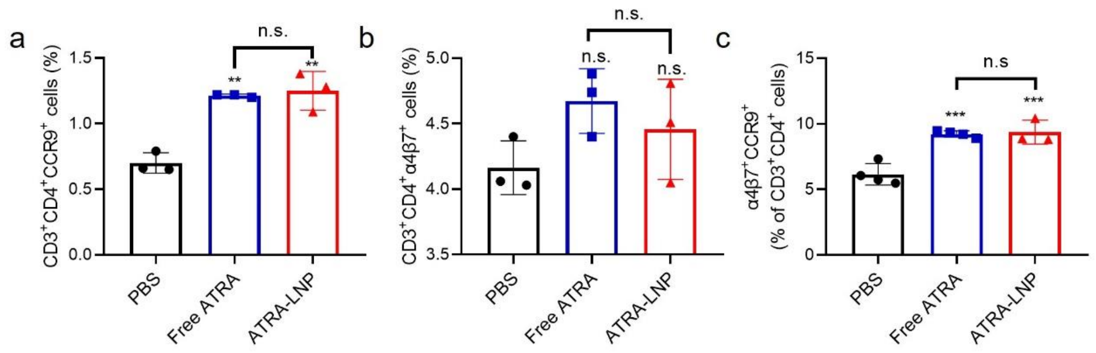

Figure S4. Spleen lymphocytes were activated by anti-CD3/CD28 antibodies-coated beads. ATRA-LNP in PBS or free ATRA in DMSO were incubated with T cells during activation. Quantification analysis of $CD3^+CD4^+CCR9^+$ (a), $CD3^+CD4^+α4β7^+$ (b), and $CCR9^+α4β7^+$ of $CD3^+CD4^+$ cells (c) among spleen lymphocytes (n = 3 or 4). Data are shown as mean ± SD. Statistical analysis was performed using one-way ANOVA and Tukey's multiple comparisons tests. **$p < 0.01$, ***$p < 0.001$, n.s. represents not statistically significant.

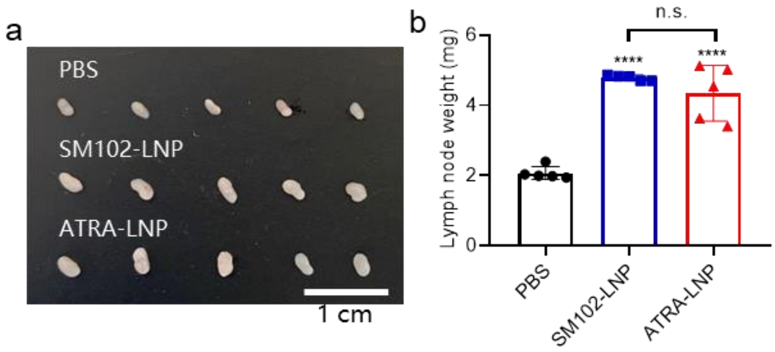

Figure S5. The draining lymph nodes were isolated 24 h post intramuscular injections with PBS, SM102-LNP, and ATRA-LNP (10 μg of mOVA per mouse). The image (a) and the weight (b) of the lymph nodes (n = 5). The scale bar is 1 cm. Each row represents five independent biological replicates. Data are shown as mean ± SD. Statistical analysis was performed using one-way ANOVA and Tukey's multiple comparisons tests. ****$p < 0.0001$, n.s. represents not statistically significant.

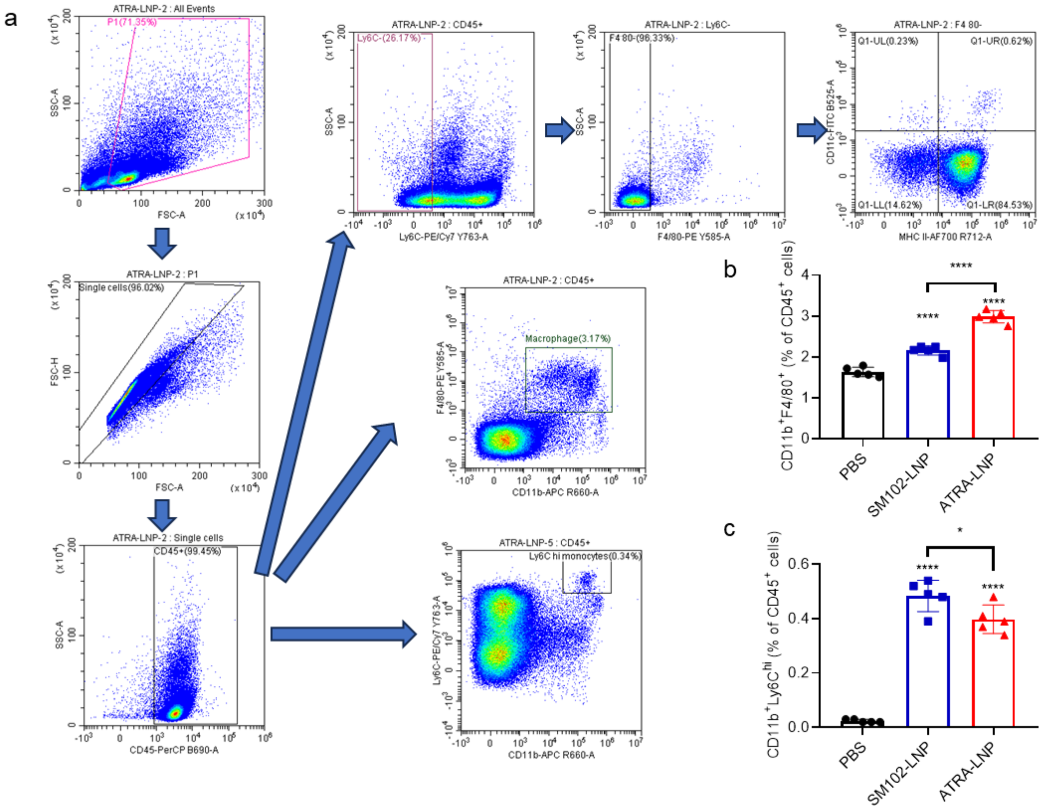

Figure S6. (a) Gating strategy for flow cytometry analysis of DCs, macrophages, and monocytes in the draining lymph node. The percentages of $CD11b^{+}F4/80^{+}$ macrophages (b) and $CD11b^{+}Ly6C^{hi}$ inflammatory monocytes (c) among $CD45^{+}$ cells in the lymph nodes (n = 5 biologically independent samples). Data are shown as mean ± SD. Statistical analysis was calculated using one-way ANOVA and Tukey's multiple comparisons tests. $*p < 0.05$, $****p < 0.0001$.

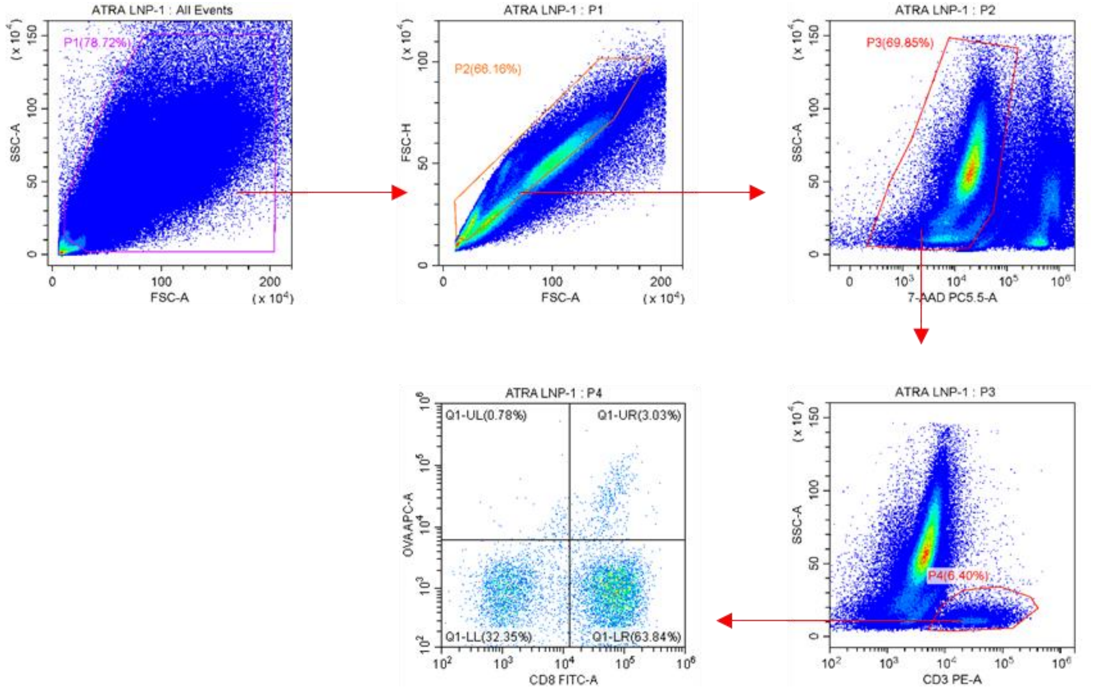

Figure S7. Gating strategy for flow cytometry analysis of antigen-specific, cytotoxic T cells in the MC38-OVA orthotopic tumor.

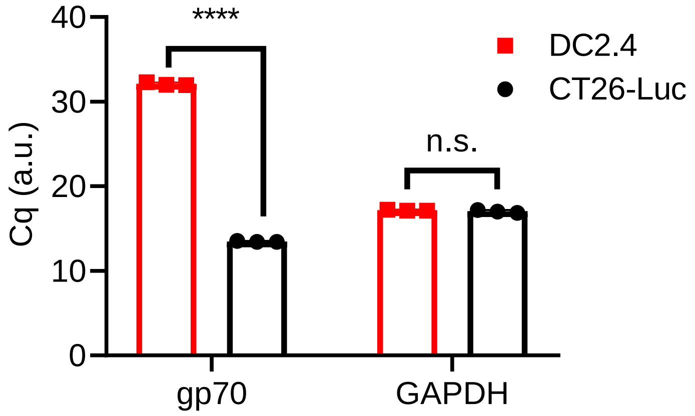

Figure S8. The amounts of gp70 mRNA in DC2.4 and CT26 cells were quantified by RT-qPCR. n = 3 technical replicates. Data are shown as mean ± SD. Statistical analysis was calculated by unpaired two-tailed Student's *t*-test. ****$p < 0.0001$, n.s. represents not statistically significant.

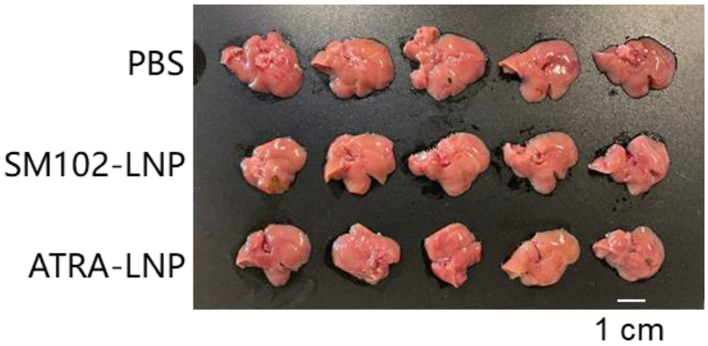

Figure S9. Optical image of livers isolated from mice bearing orthotopic CT26 tumors on days 16 post-tumor inoculation. The scale bar is 1 cm.

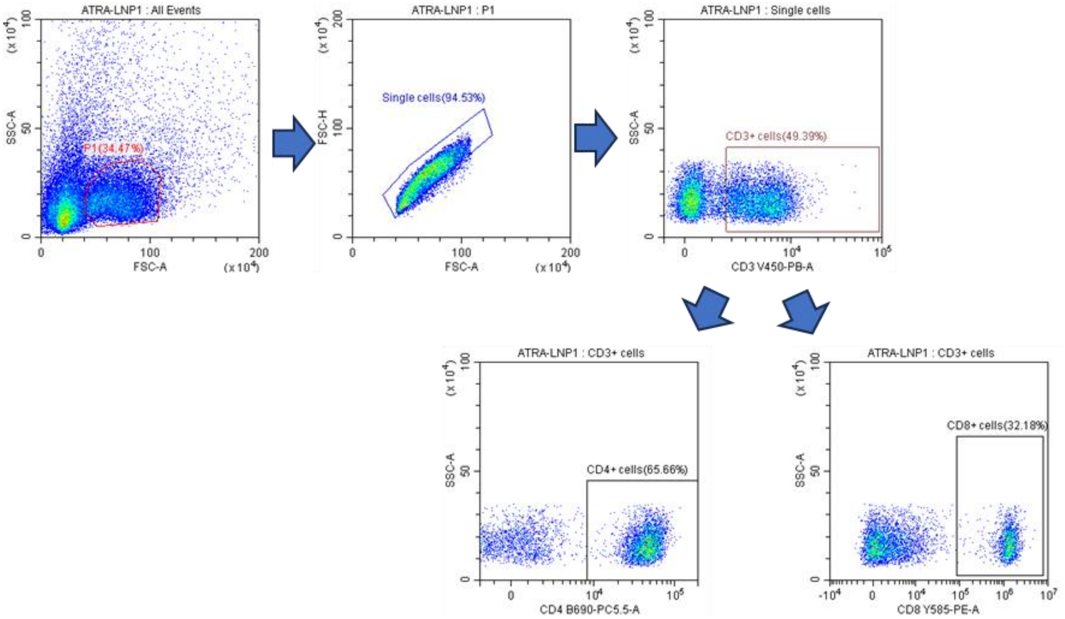

Figure S10. Gating strategy for flow cytometry analysis of $CD3^{+}CD4^{+}$ T cell and $CD3^{+}CD8^{+}$ T cell in the mesenteric lymph node of orthotopic CT26 tumor-bearing mice.

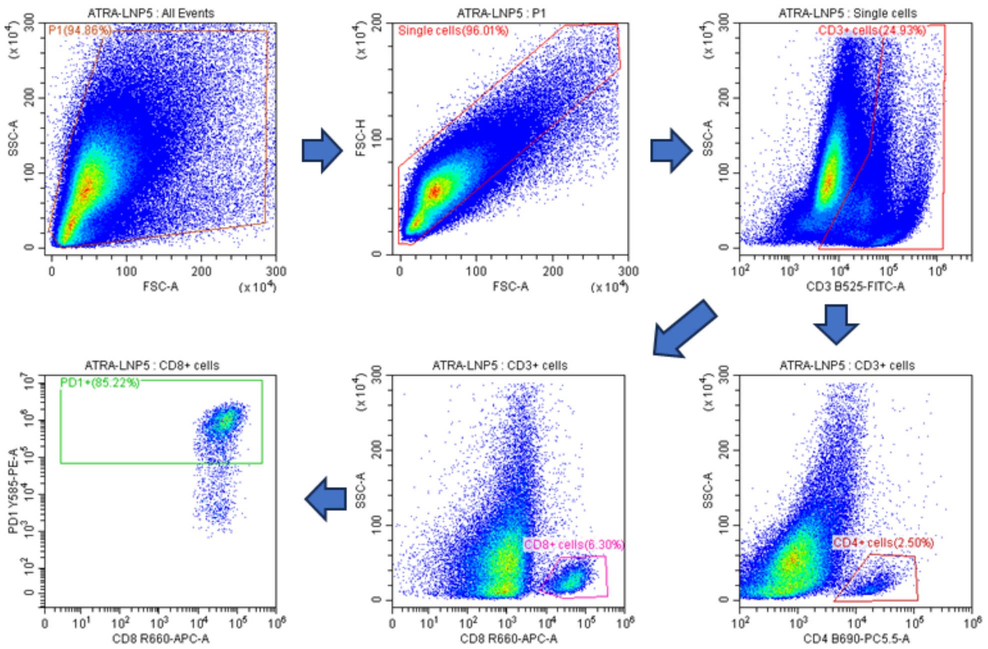

Figure S11. Gating strategy for flow cytometry analysis of $CD3^{+}CD4^{+}$ T cells, $CD3^{+}CD8^{+}$ T cells, and $CD3^{+}CD8^{+}$ $PD1^{+}$ T cells in the CT26 orthotopic tumor.

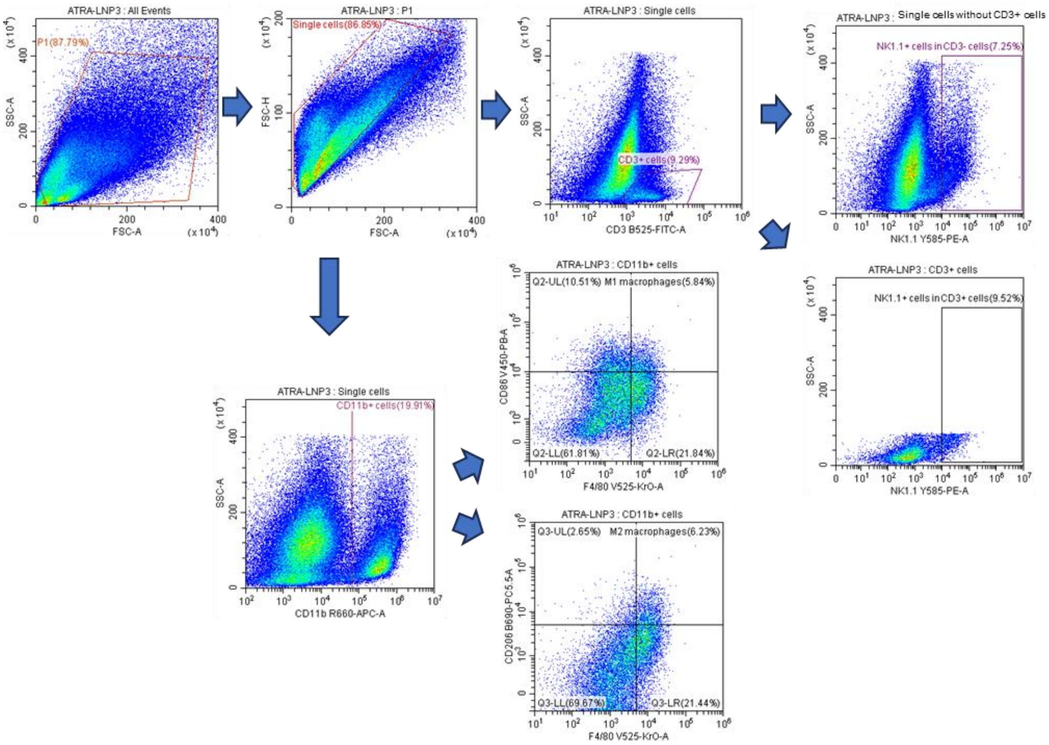

Figure S12. Gating strategy for flow cytometry analysis of CD3$^-$NK1.1$^+$ cells, CD3$^+$NK1.1$^+$ cells, CD86$^+$F4/80$^+$ cells, and CD206$^+$F4/80$^+$ in the CT26 orthotopic tumor.

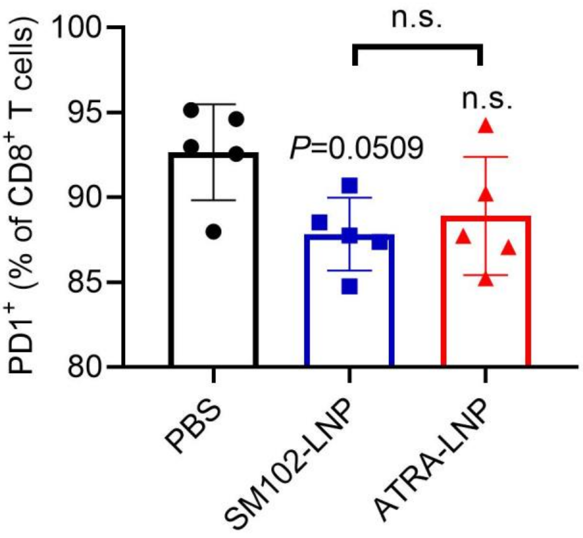

Figure S13. Percentages of PD1$^+$ cells among CD3$^+$CD8$^+$ cells in the tumor microenvironment (n = 5). Mice were injected intramuscularly with PBS, SM102-LNP, or ATRA-LNP (10 μg of gp70 mRNA per mouse) on day 1 and day 6 post-tumor inoculation. The tumors were isolated for flow cytometry on day 16 post-tumor inoculation. The vaccination schedule is depicted in Figure 7a. Data are shown as mean ± SD. Statistical analysis was performed using one-way ANOVA and Tukey's multiple comparisons tests. *$p < 0.05$, **$p < 0.01$, n.s. represents not statistically significant.

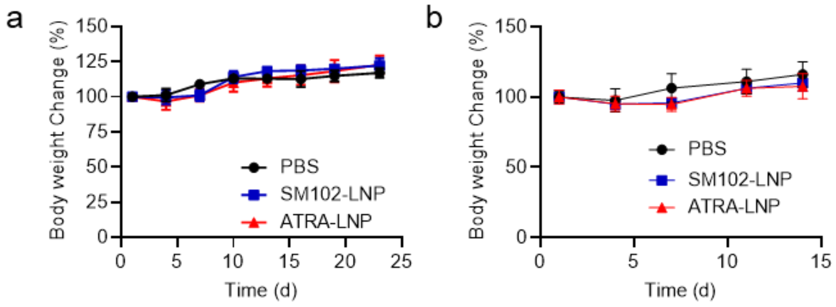

Figure S14. The body weights of mice bearing orthotopic MC38-OVA (a) or CT26 (b) were monitored following intramuscular vaccination of PBS, SM102-LNP, or ATRA-LNP (n = 6-14 biologically independent samples). Data are shown as mean ± SD.

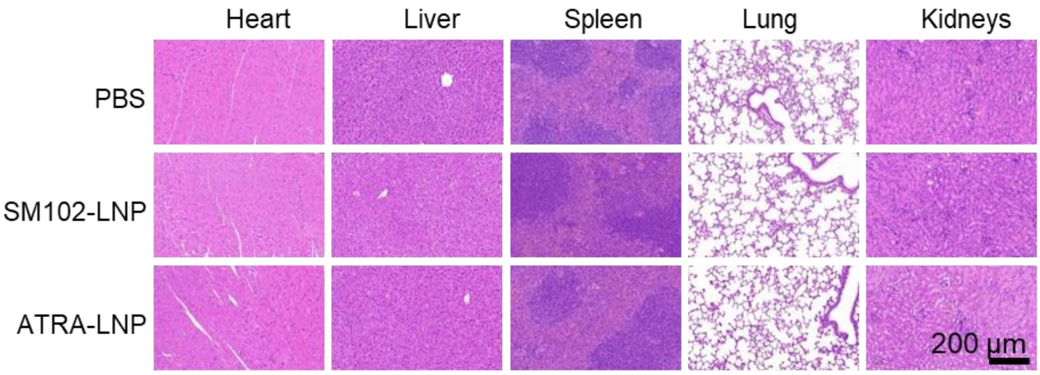

Figure S15. Mice were vaccinated through intramuscular injections with PBS, SM102-LNP, or ATRA-LNP (10 μg of mOVA per mouse) on days 1 and 6 post-tumor inoculation. Mice were sacrificed on day 23, and the major organs were extracted for H&E-stained sections. The scale bar is 200 μm.

Supplementary Table 1. mRNA encapsulation efficiency of LNPs.

Formulation	mRNA encapsulation efficiency
SM102-LNP	84.6%
ATRA-LNP (A/C=0.25)	85.7%
ATRA-LNP (A/C=0.5)	82.4%
ATRA-LNP (A/C=1)	83.3%

Supplementary Table 2. Compositions of ATRA-LNP during preparation.

Components	Molar ratio	
	N/P=5.67	N/P=15
Phosphorus in mRNA	8.8	3.3
SM102	50.0	50.0
DSPC	10.0	10.0
Cholesterol	38.5	38.5
DMG-PEG2000	1.5	1.5
ATRA	38.5	38.5
ATRA/mRNA (mass/mass)	4.1	10.8
